# Delayed Post-Hypoxic Leukoencephalopathy Following Nitrite Poisoning: A Case Report and Review of the Literature

**DOI:** 10.3389/fneur.2022.836844

**Published:** 2022-04-04

**Authors:** Yankun Chen, Qiumei Liu, Jian Wang, Hui Li, Yousheng Zhang, Lingling Sun, Jianli Liu

**Affiliations:** ^1^Department of Neurology, Heze Municipal Hospital, Heze, China; ^2^Department of Computed Tomography, Heze Municipal Hospital, Heze, China; ^3^Department of Magnetic Resonance Imaging, Heze Municipal Hospital, Heze, China; ^4^Department of Urology, Heze Municipal Hospital, Heze, China; ^5^Department of Emergency, Heze Municipal Hospital, Heze, China

**Keywords:** DPHL, leukoencephalopathy, nitrite, poisoning, case report

## Abstract

**Background:**

Delayed post-hypoxic leukoencephalopathy (DPHL) is a demyelinating syndrome that occurs days to weeks after the brain has recovered from a coma. It is caused by the period of hypoxia and is characterized by mental disorders, extrapyramidal system symptoms, and motor changes. Common causes include cardiogenic shock, severe anemia, massive blood loss, and poisoning. Poisoning, mostly resulting from intoxication with carbon monoxide and several narcotic drugs, has been reported to be a cause of DPHL. There are only a few reports of DPHL due to nitrite poisoning in literature. We report DPHL in a patient following nitrite poisoning and a review of the literature in this context.

**Case Presentation:**

A 64-year-old man presented with dizziness and nausea without vomiting. He later went into a coma after consuming a spare rib soup. After blood gas analysis, we suspected nitrite poisoning combined with metabolic acidosis, hypoxemia, and electrolyte imbalance. He gradually showed neurologic recovery to premorbid baseline after intravenous administration of methylene blue (40 mg) and symptomatic treatment. Two months later, the patient's cerebral magnetic resonance imaging (MRI) showed signs that are compatible with injury, with the patient in late stages of mental decline.

**Conclusion:**

Nitrite poisoning can cause DPHL. There is a period of intermittent recovery between the time of poisoning and the development of DPHL, but the specific pathogenesis and treatment are still unclear.

## Introduction

Nitrite, a white to yellowish powdery or granular substance similar in appearance and taste to common salt, is a common industrial reagent that has highly toxic oxidative effects. Nitrite is a food additive that serves as a color protectant or chromogenic agent (1). The nitroso compounds produced by its decomposition react with myoglobin to produce stable nitromyoglobin (bright red in color), thereby preserving the bright color of meat products (2). Nitrite can prevent the growth of *Clostridium botulinum* (3) to improve the safety of edible meat products. However, the safety range for humans of nitrite as a food additive is small. Intake of more than 0.2 g of nitrite in a single dose can provoke symptoms of poisoning in adults and is lethal at a dose of 3.0 g (4). Excessive consumption of foods in which nitrite is used as an additive will lead to the oxidation of hemoglobin, forming methemoglobin, which will hinder the release of oxygen from normal hemoglobin and result in tissue hypoxia and death. Nitrite has paralytic effects on peripheral blood vessels, resulting in symptoms such as headaches, dizziness, fatigue, chest tightness, shortness of breath, palpitations, nausea, vomiting, and cyanosis of the lips, nails, skin, and mucosal membranes. In severe cases, confusion, coma, respiratory failure, and death may occur (5).

Leukoencephalopathy can specifically refer to a large range of diseases, and the exact classification is a matter of debate. It can include progressive multifocal leukoencephalopathy, toxic leukoencephalopathy, leukoencephalopathy with vanishing white matter, leukoencephalopathy with neuroaxonal spheroids, reversible posterior leukoencephalopathy syndrome, hypertensive leukoencephalopathy, etc. Its risk factors include a genetic predisposition, metabolic syndromes, exposure to certain chemical substances (such as narcotic or antineoplastic drugs, immunosuppressants, or antibiotics) (6, 7), or carbon monoxide poisoning which leads to oxygen deficiency. Delayed post-hypoxic leukoencephalopathy (DPHL) is a rare brain demyelinating syndrome which is also known as delayed post hypoxic encephalopathy or the delayed neurological sequelae of carbon monoxide poisoning, narcotic drug overdose, or other hypoxic events. DHPL is a distinct entity separate from the secondary brain injury that occurs after these conditions and has been more closely described with toxic disorders such as carbon monoxide poisoning and drug overdoses (8–12). It is a rare brain demyelinating syndrome that can occur after any event that causes brain oxygen deficiency, such as carbon monoxide poisoning, narcotic drug overdose, myocardial infarction, or cardiopulmonary resuscitation (CPR) following cardiac arrest.

Delayed post-hypoxic leukoencephalopathy (DPHL) is characterized by a complete recovery from coma to an initially fully conscious state. Days or weeks later, it is then followed by acute onset of psychiatric disorders and neurocognitive features including disorientation, memory impairment, advanced intellectual disability, bilateral tendon hyperreflexia, frontal release signaling, and extrapyramidal manifestations, such as parkinsonism, akinetic mutism, or mental disorders. Generally, cerebral magnetic resonance imaging (MRI) shows signs compatible with diffuse bilateral demyelination of the white matter of the cerebral hemispheres not touching the cerebellum and brain stem. Cerebrospinal fluid (CSF) examination usually shows an increase in myelin basic protein.

We report DPHL in a patient following nitrite poisoning and a review of the literature in this context.

## Case Presentation

A 64-year-old man who had consumed a spare rib soup 2 h earlier presented with dizziness, nausea with the absence of vomiting, limb convulsion, and limb swelling. He was single and lived alone. When the symptoms started, he called his daughter before going into a coma. He was rushed to Mudan District Central Hospital without any special treatment to seek medical attention and was later transferred to our hospital. Upon arrival, he was in a deep coma with a Glasgow Coma Scale score (GCS) of 3. No spontaneous movements were observed. His parameters were as follows: core body temperature, 36.8°C; blood pressure, 116/65 mmHg; oxygen saturation by pulse oximetry, 80%; heart rate, 120 beats/min (tachycardia, arrhythmia, heart sound, no murmur); and respiratory rate, 26/min (tachypnea). He also presented with cyanosis of all mucous membranes and lips. The pupils were dilated and bilaterally round, ~3.0 mm in diameter, with light reflexes present. The respiratory sounds of both lungs were coarse and dry with wet rales. His abdomen was flat and soft, and no obvious enlargement of liver and spleen was observed. There was no edema in both lower extremities, low muscle tension in the limbs, no meningeal irritation, or bilateral Babinski signs.

### Supplementary Examination

No abnormally hypodensities or hyperdensities were initially found in the cerebral Computer Tomography (CT) examination, while Electrocardiogram (ECG) showed mild ST segment depression in the V1-V3 thoracic lead. Blood gas analysis produced the following values: pH, 7.39; PCO_2_, 26 mmHg; PO_2_, 67 mmHg; HCO3^−^, 15.7 mmol/l; Base Excess (BE), −7.9; Myocardial enzymes: Creatine Kinase-MB (CK-MB), 25.1 U/L; and Creatine Kinase (CK), 381.0 U/L. There were no significant abnormalities in the K^+^, Na^+^, Ca^2+^, or Cl^−^levels.

### Past Medical History

His daughter denied any history of hypertension, diabetes, coronary heart disease, close contact with people with infectious diseases, such as hepatitis or tuberculosis, trauma or surgery, or food or drug allergies of her father.

Nitrite poisoning was suspected and 20 ml of 25% glucose solution + 40 mg methylene blue 40 mg were administered by intravenous injection followed by 40 ml of 25% glucose solution +80 mg methylene blue by continuous micro-pumping. The patient gradually regained consciousness and his cognitive function was evaluated upon waking using the Montreal Cognitive Assessment (MoCA) scale (score = 27) and the Mini-Mental State Examination (MMSE) (score = 29). The coma lasted approximately 2 h before the patient regained consciousness. On the second day, a full blood count was carried out and showed a white blood cell count of 13.12 ×10^9^ /L and a neutrophil percentage of 81.7%. There were no observable abnormalities in the indicators for Acquired Immune Deficiency Syndrome (AIDS), syphilis, hepatitis C antibodies, hepatitis index, liver and renal function, and blood lipid or glucose.

After 2 days, the patient had completely recovered his mental faculties without any focal neurological deficits. Blood gas and routine blood analyses were normal, indicating that the previous increase in white blood cells was due to stress. No abnormal hypodensities or hyperdensities were found in the plain cerebral CT scan. Throughout hospitalization, he was on oxygen with ECG monitoring. We used tiopronin, cimetidine, Kudiezi, and acetylglutamine for symptomatic support treatment. Three days after admission, the patient and his family expressed great satisfaction and requested to be discharged as his condition had improved.

However, 2 months after discharge, he was readmitted to the hospital with cognitive decline and mental and behavioral abnormalities. According to family members, prior to this 2-month period, the patient had been able to communicate with people normally. They had only recently noticed that he could go a whole day without speaking a word and his reactions were slow. He used to cook every day, but he could no longer do so and behaved like a child. At times, he would hallucinate and see small animals flying on the wall. His daughter added that he was not able to clean himself properly after urinating and had difficulty walking. He would also cough intermittently during meals. Physical examination of the patient at admission showed a blood pressure of 110/68 mmHg. He had an indifferent expression and was uncooperative during physical examination, with emotional apathy. He also presented near memory loss and poor computational capacities (100–7 = ?). Both pupils appeared similar and round, with a diameter of ~3.0 mm. Light reflexes were present, limbs were movable, tendon reflexes of both lower limbs were hyperactive, and bilateral pathological symptoms were present.

**Figure 1:**
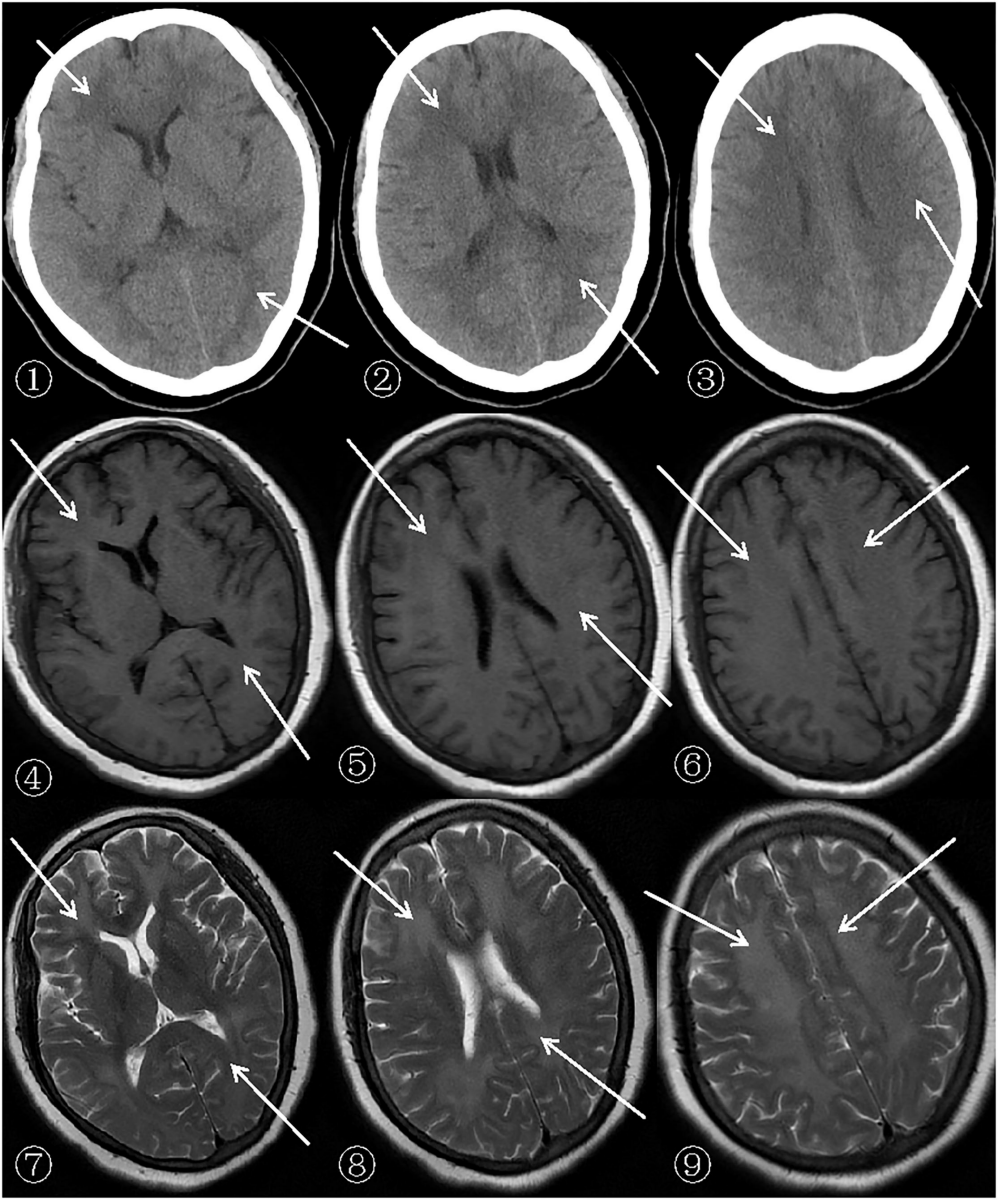
Patient's cerebral CT (General Electric Company, 64-slice Lightspeed CT) and MRI (SIEMENSAGFWB, skyra 3.0 tesla) scans.

**Figure 2:**
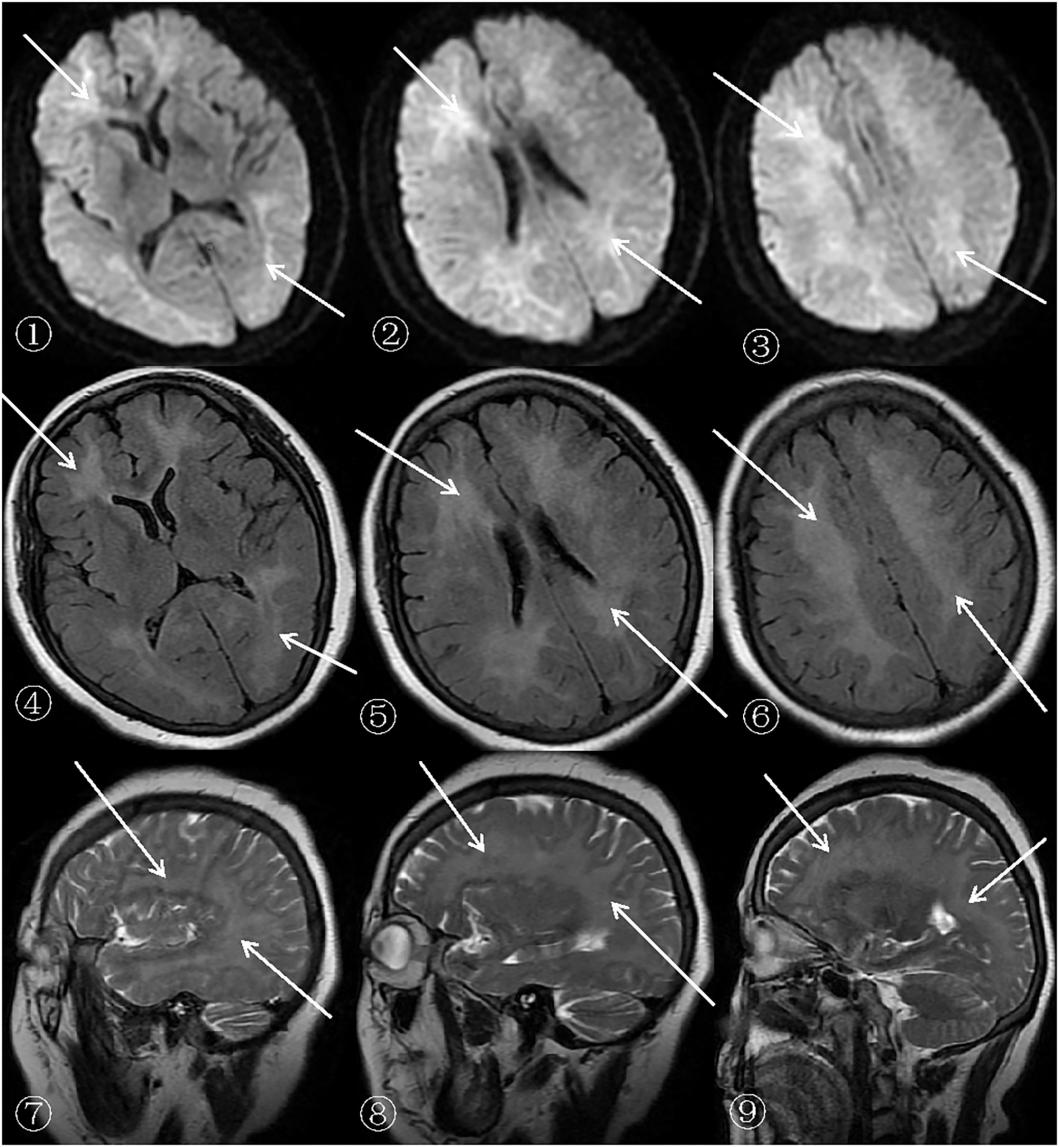
MRI (SIEMENSAGFWB, skyra 3.0 tesla) images of the patient's brain.

After admission, a cerebral CT examination was performed on the patient (presented in➀-➂ of Figure 1). Low-density shadows could be seen in the bilateral radial corona and anterior horn of the lateral ventricle. The next day, a complete cerebral MRI examination was performed (Figures 1, 2). Patchy demyelinating lesions touching the corona radiata and the centrum semiovale area were found, but the brainstem area was not affected. The ECG showed mild myocardial ischemia, while color Doppler echocardiography showed a small amount of regurgitation in the mitral and tricuspid regions. The chest X-ray indicated bronchitis. Routine blood analysis showed a white blood cell count of 14.8 × 10^9^/L and a neutrophil percentage of 85.7%. The clinical symptoms of sepsis were not serious and the patient was afebrile with cough (no expectorant, chest tightness, or wheezing). Blood gas analysis indicated a pH of 7.42, PCO_2_ of 32 mmHg, and PO_2_ of 90 mmHg. Sputum was collected for culture after admission, and *Klebsiella pneumoniae*, which was sensitive to ceftriaxone sodium according to the drug sensitivity test, was found. His septic symptoms greatly improved after 14 days of treatment with ceftriaxone sodium. Routine blood analysis was normal and the chest radiograph showed marked improvement in bronchitis. CSF analysis showed increased levels of the myelin sheath basic protein (MBP), with cytology and pressure within normal ranges. Cognitive function was evaluated using MoCA (score = 5) and MMSE (score = 7). Despite treatment, his symptoms did not improve even after 14 days of hospitalization. On demand by the patient's family, he was discharged even though he still had slow reactions and was functionally dependent. According to the 6-month follow-up, he was still functionally dependent and had been living under the care of his daughter.

The scan shows bilateral diffuse low density white matter in the anteroposterior horn of the lateral ventricle, basal ganglia, and thalamus. MRI of the cerebral showed abnormally long T1 (presented in ➃-➅ of Figure 1) and T2 (presented in ➆-➈ of Figure 1) signals in the white matter around the bilateral corona radiata, semiovale center, and lateral ventricle, with fluid attenuated inversion recovery (FLAIR) (presented in➃-➅ of Figure 2) showing bright signals. Sagittal images: T2 (presented in ➆-➈ of Figure 2) showed a large number of hyperintense lesions around the lateral ventricle, but the cerebral cortex, U-fibers, cerebellum, and brain stem are not affected.

There was a diffusely increased signal intensity in the bilateral cerebral white matter. Diffusion weighted imaging (DWI) (presented in ➀-➂ of Figure 2) showing areas of diffuse and symmetric diffusion restriction in the bilateral cerebral subcortical white matter that correspond to the changes observed in the FLAIR (presented in ➃-➅ of Figure 2) images.

## Discussion

Delayed post-hypoxic leukoencephalopathy (DPHL) is a demyelinating syndrome that occurs days to weeks after the brain has recovered from a coma caused by a period of hypoxia. It is characterized by acute neuropsychiatric symptoms such as mental disorders, extrapyramidal system symptoms, and pyramidal tract syndrome. Common causes include cardiogenic shock, severe anemia, cardiopulmonary arrest, shock, (13, 14) and metabolic comas such as hepatic, hyperglycemic, or hypoglycemic comas (15). Clinically, it is characterized by the patient's return to a normal state after recovery from the coma and subsequent respiratory arrest (10, 11, 16, 17). In the present case, the patient had normal brain imaging examination results at admission and was discharged after symptoms had improved. After a seemingly normal recovery, he was readmitted to hospital for symptoms of diffuse leukoencephalopathy. Such diffuse leukoencephalopathy typically appears shortly after an initial short recovery period (14–16, 18). Severe cognitive and behavioral changes, such as eccentric behavior, personality changes, confusion, memory disorders, mutism, hemiplegia, tension, consciousness disorders, incontinence, and abnormal movements are also symptoms of this condition (11–13, 17, 19, 20). Routine CSF cell count and protein and glucose levels in patients with DPHL are usually normal and microbial culture is usually negative, with no evidence of infection. In addition, oligoclonal bands usually are not present and CSF IgG levels are usually normal. However, there have been reports of cases with positive cerebrospinal fluid myelin protein (10, 11). With this condition, electroencephalogram (EEG) might be able to reveal diffuse and generalized slow wave activity (10, 13, 19, 21). The cerebral CT and MRI scans showed diffuse white matter lesions on both sides of the brain. The cortex, putamen, thalamus, hippocampus, brainstem, and cerebellum were not affected (10). During hypoxia, which leads to ischemia, the body must first ensures cardio-cerebral blood supply. Blood supply to the lungs, kidneys, skin, and other organs will decline, but as the oxygen demands of the body increase over time, there is loss of this compensatory mechanism. In the end, blood flow to the brain decreases due to heart failure and systemic blood pressure drops, leading to brain blood flow redistribution. First, blood flow is ensured to those parts of the brain with the highest metabolism, such as the basal ganglia and thalamus, cerebellum, and brainstem, with a significant reduction in blood supply to the subcortical white matter, leading to bilateral damage to the lateral ventricles. If ischemia and hypoxia are acute, the parts of brain with the highest metabolism, such as the basal ganglia, are significantly damaged (22–24). During carbon monoxide poisoning, due to the local anemia and hypoxia, the oxygen free radicals and peroxides in the brain cells and lipid peroxidation of the cell membrane increases, leading to the demyelination of nerve cells (25). In addition, Nabeshima et al. (20, 26) found that carbon monoxide poisoning may lead to changes in the structure or function of the myelin phospholipid protein, abnormal expression of the axon growth regulatory factor, and an unbalanced expression of some cytokines and inflammatory molecules in the central nervous system, all of which are important factors that lead to the demyelination of brain white matter (27, 28). However, the pathophysiology of delayed hypoxic leukoencephalopathy, with an intermediate period of false healing, has not yet been clearly understood. Heckmann et al. (23) attributed the process of delayed hypoxic leukoencephalopathy to slow myelin necrosis. Because the half-life of the myelin sheath is longer, the glial cells that produce myeloid cells in the white matter boundary area immediately die, and the white matter perfusion becomes less than that of gray matter. Another possible explanation is that the myelin forming cells in the white matter are stimulated into a sensitive state, originating from the mitochondrial release of cytochrome C and associated stimulation of caspase-3 or the activation of cell surface death receptors, resulting in the stimulation of caspase-8 (29) and the decrease in the levels of arylsulfatase A (12).

The patient may have had hereditary leukodystrophy. However, Salman et al. (30) postulated that long-term hypoxia, delayed resuscitation, and/or multiple organ failure and hypoperfusion may be risk factors for early development of leukoencephalopathy, especially in patients with drug overdose. These patients usually have severe clinical manifestations and poor prognosis.

At present, there are no clear diagnostic criteria for DPHL. It is usually diagnosed based on a history of hypoxia and the exclusion of other encephalopathy-causing disease using the results of head imaging (31). Careful inquiry into the medical history, examination of corresponding clinical workups (such as liver and kidney function, blood gas analysis, thyroid function, homocysteine levels, methylmalonic acid levels, blood electrolytes, routine CSF analysis, full blood count, oligoclonal bands, AQP4 antibodies, smear, and culture of pathogenic microorganisms), and EEG and head imaging examinations are of great importance for a differential diagnosis. The limitation in this case is that the relationship of DPHL to nitrite poisoning is only speculative and not conclusive. Upon readmission, the patient still had signs of myocardial ischemia and bronchitis. These contradictions may have contributed, to some degree, to the patient's readmission and development of DPHL. However, it seems unlikely that there is a direct causal relation between these pathologic conditions and the DPHL (32, 33). Hence, we do not think they cause DPHL. Since the patient denied the previous history of coronary heart disease, we considered that myocardial ischemia might also be caused by nitrate poisoning. As discussed beforehand, it is postulated that DPHL was caused by nitrite poisoning. Hence, the patient's DPHL might be caused by this food additive, requiring awareness of the possibility and attention to food safety.

After a period of intermittent recovery following nitrite poisoning, a series of neurological deficit symptoms gradually appeared. The cerebral MRI showed extensive demyelination in the white matter of the cerebral hemispheres, which is similar to that observed in patients with DPHL after acute carbon monoxide poisoning. The characteristics of the patient we reported here are consistent with those of patients with leukoencephalopathy after acute carbon monoxide poisoning. At present, the pathogenesis of DPHL caused by nitrite poisoning is not clear. It is possibly due to the fact that nitrite oxidizes the ferrous iron in hemoglobin to trivalent iron. This transforms hemoglobin to methemoglobin and causes it to lose its oxygen-carrying capacity, leading to tissue hypoxia. As the central nervous system is more sensitive to hypoxia, vasospasms, degeneration of the vascular wall cells, vascular dilatation, congestion, vascular rupture, and occlusive endarteritis appear first, followed by the degeneration of nerve cells, glial cell proliferation, brain tissue necrosis, and softening. Due to the relatively few capillaries in white matter, it is more likely to be damaged during a period of hypoxia, resulting in extensive myelin sheath loss and delayed encephalopathy (34).

No single treatment has been shown to be effective in the first 2 weeks of the onset of the neuropsychiatric symptoms of DPHL. It is mainly managed through symptomatic supportive therapies, such as antispasmodic measures, ensuring adequate energy and oxygen supply, and maintaining water, electrolyte, and acid-base balance. Neither high doses of methylprednisolone nor coenzyme Q have been shown to have significant therapeutic effects (12, 30). There is no effective treatment to reverse its rapid progression into coma. Targeted Temperature Management (TTM), as a neuroprotective treatment, has not been systematically studied in the context of DPHL, but may represent an important measure to improve the neurological outcome. It may also, perhaps, prevent the development of DPHL. Hence, further studies are needed to clarify the therapeutic role of TTM in patients at risk of DPHL.

Immunotherapy (steroid and plasma exchange) has been tested in other countries with unsatisfactory results (31). In China, Yankun et al. (35) carried out a study on 171 patients with leukoencephalopathy after carbon monoxide poisoning. After the administration of a butylphthalide injection in combination with butylphthalide soft capsules and low-dose dexamethasone combined with hyperbaric oxygen treatment in the comprehensive treatment group, the cognitive and motor functions of patients significantly improved. This study provides insight into the treatment of delayed leukoencephalopathy after hypoxia. Rehabilitation is also an integral part of the treatment of leukoencephalopathy. Once patients are evaluated to be ready, rehabilitation treatment should be started immediately. Physical, occupational, language, and entertainment therapies are carried out in patients with specific nerve injuries to evaluate their functional status and utilize the information obtained from the nerve rehabilitation assessment to formulate treatment goals (31).

Most patients with DPHL recover slowly (30). The disease has peculiar clinical and imaging manifestations. Symptoms of delayed encephalopathy after acute hypoxia, cerebral MRI imaging showing white matter disease, and elevated myelin basic protein in the CSF all indicate DPHL. Most patients usually recover completely. However, DPHL can easily induce long-term neurological deficits. Within a few weeks, patients with mild symptoms gradually begin to recover some functions, such as speaking and walking. After 1–−2 years, most patients return to their baseline level, but following such poisoning, cognitive impairment including short-term memory deficits and fatigue may persist and hamper the patient's independence, reducing their quality of life (21, 36–39). This calls for heightened awareness of DPHL as a differential diagnosis of the pathogenesis of leukoencephalopathy.

A written informed consent was obtained from the patient's daughter for publication of this case report and any accompanying images. The written consent is available for review by the editor of this esteemed journal.

## Data Availability Statement

The original contributions presented in the study are included in the article/supplementary material, further inquiries can be directed to the corresponding author.

## Ethics Statement

Written informed consent was obtained from the individual's legal guardian/next of kin for the publication of any potentially identifiable images or data included in this article.

## Author Contributions

YC, YZ, and JL contributed to conception and design of the study. QL, JW, and LS organized the databases and the figures. YC wrote the first draft of the manuscript. LS, HL, and QL wrote sections of the manuscript. All authors contributed to manuscript revision and approved the submitted version.

## Conflict of Interest

The authors declare that the research was conducted in the absence of any commercial or financial relationships that could be construed as a potential conflict of interest.

## Publisher's Note

All claims expressed in this article are solely those of the authors and do not necessarily represent those of their affiliated organizations, or those of the publisher, the editors and the reviewers. Any product that may be evaluated in this article, or claim that may be made by its manufacturer, is not guaranteed or endorsed by the publisher.

## References

[1] Cvetković D Živković V Lukić V Nikolić S. Sodium nitrite food poisoning in one family. Forensic Sci Med Pathol. (2019) 15:102–5. 10.1007/s12024-018-0036-1 30293223

[2] Institute of American Meat Packers. Sausage and ready to serve meats. Chicago, Institute of Meat Packing, Haskell Hall, University of Chicago. (1938).

[3] Krause BL Sebranek JG Rust RE Mendonca A. Incubation of curing brines for the production of ready-to-eat, uncured, no-nitrite-or-nitrate-added, ground, cooked and sliced ham. Meat Sci. (2011) 89:507–13. 10.1016/j.meatsci.2011.05.018 21664056

[4] Wright RO Lewander WJ Woolf AD. Methemoglobinemia: etiology, pharmacology, and clinical management. Ann Emerg Med. (1999) 34:646–56. 10.1016/S0196-0644(99)70167-8 10533013

[5] Doctor L. The classification and treatment of methemoglobinemia. Q Bull Northwest Univ Med Sch. (1953) 27:134–7. 13056142 PMC3803312

[6] Filley CM Kleinschmidt-DeMasters BK. Toxic leukoencephalopathy. N Engl J Med. (2001) 345:425–32. 10.1056/NEJM200108093450606 11496854

[7] Krinsky CS Ross RR. Chasing the dragon: a review of toxic leukoencephalopathy. Acad Forensic Pathol. (2012) 2:67–73. 10.23907/2012.009

[8] Sioka C Kyritsis AP. Central and peripheral nervous system toxicity of common chemotherapeutic agents. Cancer Chemother Pharmacol. (2009) 63:761–7. 10.1007/s00280-008-0876-6 19034447

[9] Kim JH Chang KH Song IC Kim KH Kwon BJ Kim HC. Delayed encephalopathy of acute carbon monoxide intoxication: diffusivity of cerebral white matter lesions. AJNR Am J Neuroradiol. (2003) 24:1592–7. 13679276 PMC7973971

[10] Zamora CA Nauen D Hynecek R Ilica AT Izbudak I Sair HI. Delayed posthypoxic leukoencephalopathy: a case series and review of the literature. Brain Behav. (2015) 5:e00364. 10.1002/brb3.364 26357591 PMC4559021

[11] Chang WL Chang YK Hsu SY Lin GJ Chen SC. Reversible delayed leukoencephalopathy after heroin intoxication with hypoxia:a case report. Acta Neurol Taiwan. (2009) 18:198–202. 19960964

[12] Gottfried JA Mayer SA Shungu DC Chang Y Duyn JH. Delayed posthypoxic demyelination. Association with arylsulfatase A deficiency and lactic acidosis on proton MR spectroscopy. Neurology. (1997) 49:1400–4. 10.1212/WNL.49.5.1400 9371929

[13] Rizzuto N Morbin M Ferrari S Cavallaro T Sparaco M Boso G. Delayed spongiform leukoencephalopathy after heroin abuse. Acta Neuropathol. (1997) 94:87–90. 10.1007/s004010050676 9224535

[14] B Bartlett E Mikulis DJ. Chasing “chasing the dragon” with MRI: leukoencephalopathy in drug abuse. Br J Radiol. (2005) 78:997–1004. 10.1259/bjr/61535842 16249600

[15] Ginsberg MD. Delayed neurological deterioration following hypoxia. Adv Neurol. (1979) 26:21–44. 517295

[16] McKinney AM Kieffer SA Paylor RT SantaCruz KS Kendi A Lucato L. Acute toxic leukoencephalopathy: potential for reversibility clinically and on MRI with diffusion-weighted and FLAIR imaging. AJR Am J Roentgenol. (2009) 193:192–206. 10.2214/AJR.08.1176 19542414

[17] Chen-Plotkin AS Pau KT Schmahmann JD. Delayed leukoencephalopathy after hypoxic-ischemic injury. Arch Neurol. (2008) 65:144–5. 10.1001/archneurol.2007.7 18195154

[18] Ryan A Molloy FM Farrell MA Hutchinson M. Fatal toxic leukoencephalopathy: clinical, radiological, and necropsy findings in two patients. J Neurol Neurosurg Psychiatry. (2005) 76:1014–6. 10.1136/jnnp.2004.047134 15965216 PMC1739717

[19] Lee HB Lyketsos CG. Delayed post-hypoxic leukoencephalopathy. Psychosomatics. (2001) 42:530–3. 10.1176/appi.psy.42.6.530 11815692

[20] Wallace IR Dynan C Esmonde T. One confused patient, many confused physicians: a case of delayed post-hypoxic leucoencephalopathy. QJM. (2010) 103:193–4. 10.1093/qjmed/hcp155 19864347

[21] Shprecher DR Flanigan KM Smith AG Smith SM Schenkenberg T Steffens J. Clinical and diagnostic features of delayed hypoxic leukoencephalopathy. J Neuropsychiatry Clin Neurosci. (2008) 20:473–7. 10.1176/jnp.2008.20.4.473 19196933

[22] Roychowdhury S Maldjian JA Galetta SL Grossman RI. Postanoxic encephalopathy: diffusion MR findings. J Comput Assist Tomogr. (1998) 22:992–4. 10.1097/00004728-199811000-00028 9843246

[23] Heckmann JG Erbguth F Neundörfer B. Delayed postanoxic demyelination registry. Neurology. (1998) 51:1235–6. 10.1212/WNL.51.4.1235-b 9781585

[24] White BC Grossman LI Krause GS. Brain injury by global ischemia and reperfusion: a theoretical perspective on membrane damage and repair. Neurology. (1993) 43:1656–65. 10.1212/WNL.43.9.1656 8414008

[25] Gorman D Drewry A Huang YL Sames C. The clinical toxicology of carbon monoxide. Toxicology. (2003) 187:25–38. 10.1016/S0300-483X(03)00005-2 12679050

[26] Nabeshima T Katoh A Ishimaru H. Carbon monoxide-induced delayed amnesia, delayed neuronal death and change in acetylcholine concentration in mice. J Pharmacol Exp Ther. (1991) 256:378–84. 1671097

[27] Li Q Cheng Y Bi MJ Kang H Qu Y Lin H. Effects of N-Butylphthalide on the expressions of Nogo/NgR in rat brain tissue after carbon monoxide poisoning. Environ Toxicol Pharmacol. (2015) 39:953–61. 10.1016/j.etap.2015.02.013 25812770

[28] Yang Y Liu Y Wei P Peng H Winger R Hussain RZ. Silencing Nogo-A promotes functional recovery in demyelinating disease. Ann Neurol. (2010) 67:498–507. 10.1002/ana.21935 20437585 PMC2929680

[29] Broughton BR Reutens DC Sobey CG. Apoptotic mechanisms after cerebral ischemia. Stroke. (2009) 40:e331–9. 10.1161/STROKEAHA.108.531632 19182083

[30] Aljarallah S Al-Hussain F. Acute fatal posthypoxic leukoencephalopathy following benzodiazepine overdose:a case report and review of the literature. BMC Neurol. (2015) 15:69. 10.1186/s12883-015-0320-6 25925073 PMC4418099

[31] Shprecher D Mehta L. The syndrome of delayed post-hypoxic leukoencephalopathy. NeuroRehabilitation. (2010) 26:65–72. 10.3233/NRE-2010-0536 20166270 PMC2835522

[32] Boerlin A Luescher T Becker C Perrig S Thommen E Widmer M. Low Plasma Sphingomyelin Levels Show a Weak Association with Poor Neurological Outcome in Cardiac Arrest Patients: Results from the Prospective, Observational COMMUNICATE Trial. J Clin Med. (2020) 9:897. 10.3390/jcm9040897 32218134 PMC7230482

[33] Isenschmid C Luescher T Rasiah R Kalt J Tondorf T Gamp M. Performance of clinical risk scores to predict mortality and neurological outcome in cardiac arrest patients. Resuscitation. (2019) 136:21–9. 10.1016/j.resuscitation.2018.10.022 30391369

[34] Xinhong F Zhonghui Z Shimeng Z. A case of delayed encephalopathy after nitrite poisoning. Chin J. Neuroimmunol Neurol. (2013) 20:343.

[35] Zhang J Guo Y Li W Li G Chen Y. The Efficacy of N-Butylphthalide and Dexamethasone Combined with Hyperbaric Oxygen on Delayed Encephalopathy After Acute Carbon Monoxide Poisoning. Drug Des Devel Ther. (2020) 14:1333–9. 10.2147/DDDT.S217010 32308366 PMC7135188

[36] Choi IS. Delayed neurologic sequelae in carbon monoxide intoxication. Arch Neurol. (1983) 40:433–5. 10.1001/archneur.1983.04050070063016 6860181

[37] Barnett MH Miller LA Reddel SW Davies L. Reversible delayed leukoencephalopathy following intravenous heroin overdose. J Clin Neurosci. (2001) 8:165–7. 10.1054/jocn.2000.0769 11243768

[38] Arciniegas DB Frey KL Anderson CA Brousseau KM Harris SN. Amantadine for neurobehavioural deficits following delayed post-hypoxic encephalopathy. Brain Inj. (2004) 18:1309–18. 10.1080/02699050410001720130 15666573

[39] Lam SP Fong SY Kwok A Wong T Wing YK. Delayed neuropsychiatric impairment after carbon monoxide poisoning from burning charcoal. Hong Kong Med J. (2004) 10:428–31. 15591604

